# Exploring Bioactive Properties of Marine Cyanobacteria Isolated from the Portuguese Coast: High Potential as a Source of Anticancer Compounds

**DOI:** 10.3390/md12010098

**Published:** 2013-12-31

**Authors:** Margarida Costa, Mónica Garcia, João Costa-Rodrigues, Maria Sofia Costa, Maria João Ribeiro, Maria Helena Fernandes, Piedade Barros, Aldo Barreiro, Vitor Vasconcelos, Rosário Martins

**Affiliations:** 1 Interdisciplinary Center of Marine and Environmental Research (CIIMAR/CIMAR), University of Porto, Rua dos Bragas 289, Porto 4050-123, Portugal; E-Mails: costa.anamarg@gmail.com (M.C.); marysofs@gmail.com (M.S.C.); maria.joaox@hotmail.com (M.J.R.); aldo.barreiro@gmail.com (A.B.); vmvascon@fc.up.pt (V.V.); 2 Laboratory for Bone Metabolism and Regeneration, Faculty of Dental Medicine, University of Porto, Rua Dr. Manuel Pereira da Silva, Porto 4200-393, Portugal; E-Mails: mgarcia@fmd.up.pt (M.G.); jrodrigues@fmd.up.pt (J.C.-R.); mhfernandes@fmd.up.pt (M.H.F.); 3 Health and Environmental Research Center (CISA), Superior School of Health Technology of Porto, Polytechnic Institute of Porto, Rua Valente Perfeito 322, Vila Nova de Gaia 4400-330, Portugal; E-Mail: pgb@estsp.ipp.pt; 4 Department of Biology Faculty of Sciences, University of Porto, Rua do Campo Alegre, Edifício FC4, Porto 4169-007, Portugal; 5 IBMC (Institute for Molecular and Cell Biology), University of Porto, Rua do Campo Alegre 823, Porto 4150-180, Portugal

**Keywords:** marine cyanobacteria, natural products, anticancer potential

## Abstract

The oceans remain a major source of natural compounds with potential in pharmacology. In particular, during the last few decades, marine cyanobacteria have been in focus as producers of interesting bioactive compounds, especially for the treatment of cancer. In this study, the anticancer potential of extracts from twenty eight marine cyanobacteria strains, belonging to the underexplored picoplanktonic genera, *Cyanobium*, *Synechocystis* and *Synechococcus*, and the filamentous genera, *Nodosilinea*, *Leptolyngbya*, *Pseudanabaena* and *Romeria*, were assessed in eight human tumor cell lines. First, a crude extract was obtained by dichloromethane:methanol extraction, and from it, three fractions were separated in a Si column chromatography. The crude extract and fractions were tested in eight human cancer cell lines for cell viability/toxicity, accessed with the 3-(4,5-dimethylthiazol-2-yl)-2,5-diphenyl tetrazolium bromide (MTT) and lactic dehydrogenase release (LDH) assays. Eight point nine percent of the strains revealed strong cytotoxicity; 17.8% showed moderate cytotoxicity, and 14.3% assays showed low toxicity. The results obtained revealed that the studied genera of marine cyanobacteria are a promising source of novel compounds with potential anticancer activity and highlight the interest in also exploring the smaller filamentous and picoplanktonic genera of cyanobacteria.

## 1. Introduction

Exploring the potential bioactivities of organisms is still a remarkable tool for the development of new pharmacological products [1]. Despite the progresses in drug synthesis, screening natural compounds directly from the producer organism still provides a high percentage of new compounds for clinical trials [2].

The ocean hosts an unmeasured biological and chemical diversity, which led to an increased research effort in natural products. Marine cyanobacteria, in particular, have become a promising source of new compounds with interest in pharmacology and biotechnology [3]. Despite their relative simplicity, cyanobacteria are spread through the whole range of marine environments, revealing a high capacity of adaptation. Concerning the production of bioactive compounds, a single cyanobacterial strain is capable of producing an array of secondary metabolites with distinct chemical arrangements and interesting bioactivities. As an example, a *Lyngbya majuscula* strain collected in Grenada was found to produce two new halogenated fatty acid amides (grenadamides B and C), two depsipeptides, (itralamides A and B) and two lipopeptides (hectochlorin and deacetylhectochlorin) [4].

**Figure 1:**
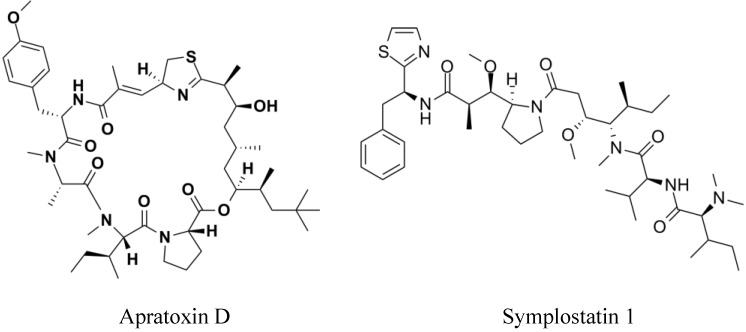
Chemical structures of the marine cyanobacteria secondary metabolites, apratoxin D and symplostatin 1.

As a result of exploring cyanobacteria for bioactive natural products, many compounds were described, and in bioassay-guided fractionation screenings, many revealed antibacterial [5], antimalarial [6] and anti-inflammatory [7] activities. However, despite the large array of bioactivities of the compounds, researchers have focused on their potential as anticancer drugs. Several compounds isolated from marine cyanobacteria demonstrated strong cytotoxicity against human tumor cells [8,9,10]. Apratoxin D (Figure 1), from *Lyngbya majuscula* and *Lyngbya sordida* collections, showed an IC_50_ value of 2.6 nM against H-460 human lung cancer cells [9]. Symplostatin 1 (Figure 1), isolated from a *Symploca hydnoides* strain, induces cell death in the MDA-MB-435 breast carcinoma cell line with an IC_50_ of 0.15 nM and in NCI/ADR ovarian carcinoma cells with an IC_50_ of 0.09 nM [11].

The cytotoxicity of cyanobacterial natural compounds in cancer cell lines is induced by different mechanisms. Coibamide, a cyclic depsipeptide, isolated from a Panamanian *Leptolyngbya* sp. strain, causes cell cycle arrest in the G_1_ phase in MDA-MB-435 breast cancer cells [12]. Bouillomides A and B, another two depsipeptides isolated from *Lyngbya bouillonii*, were found to specifically and strongly inhibit serine proteases elastase and trypsin [13]. Other marine cyanobacteria compounds are capable of inducing oxidative stress and DNA fragmentation, microfilament disruption, Bcl-2 protein family modulation and even alterations in cell membrane dynamics [14,15,16,17].

The majority of the described natural compounds produced by marine cyanobacteria were isolated from filamentous species that grow in large densities along the shores and, consequently, are easy to collect [18]. Among these cyanobacteria, the genus *Lyngbya*, or *Moorea* for some strains, as recently found [19,20], has been the most prolific. We believe that the higher success of these filamentous cyanobacteria is due to a biased research effort. This bias seems to be due to an easier collection of biomass from these cyanobacteria in the field. Due to their slower growth in environmental conditions, other cyanobacteria groups, such as the picoplanktonic *Cyanobium*, *Synechocystis* and *Synechococcus* and the filamentous *Nodosilinea*, *Leptolyngbya*, *Pseudanabaena* and *Romeria*, have been largely overlooked. These cyanobacteria genera constitute a large fraction of the marine cyanobacterial strains isolated from the Portuguese coast and maintained in the culture collection of our research institution. In the present study, we aimed to evaluate the bioactive potential of strains belonging to these genera, by screening their cytotoxicity in human cancer lines. A crude extract from twenty eight cyanobacteria strains was obtained by dichloromethane:methanol extraction. This extract was further fractionated into three fractions, in Si column chromatography. Fractions A, B and C, were eluted according to their polarity with a stepped gradient from 100% hexane, 100% ethyl acetate and 100% methanol. Cell toxicity was evaluated on eight cancer cell lines by 3-(4,5-dimethylthiazol-2-yl)-2,5-diphenyl tetrazolium bromide assay (MTT). The stronger positive results from MTT were tested by the lactic dehydrogenase release assay (LDH), in order to select the most promising strains for the further isolation of bioactive compounds.

## 2. Results

**Figure 2:**
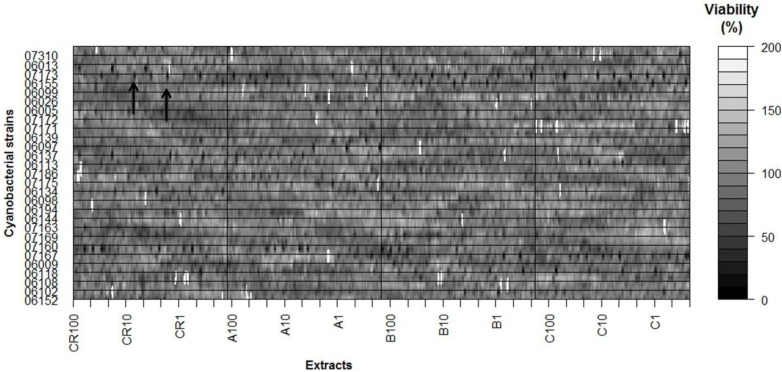
Results of all individual experiments of the MTT assay. The percentage of cell viability was calculated relative to the control. Dark dots (arrows indicate examples) are indicative of strong and moderate cytotoxicity (CR100, 10 and 1: crude extract at 100, 10 and 1 μg·mL^−1^; A100, 10 and 1: fraction A at 100, 10 and 1 μg·mL^−1^; B100, 10 and 1: fraction B at 100, 10 and 1 μg·mL^−1^; C100, 10 and 1: fraction C at 100, 10 and 1 μg·mL^−1^).

**Figure 3:**
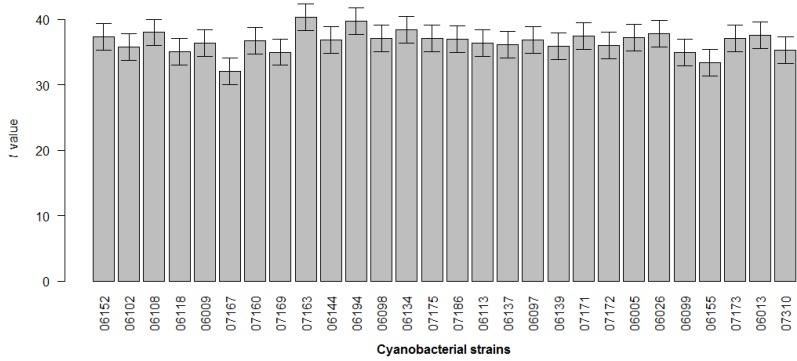
Global toxicity of cyanobacterial strains. The average effect of strains on cell viability was calculated with respect to the positive control and standardized with the *t* statistic (*n* ~ 857). Error bars show the standard error of the estimated difference.

**Figure 4:**
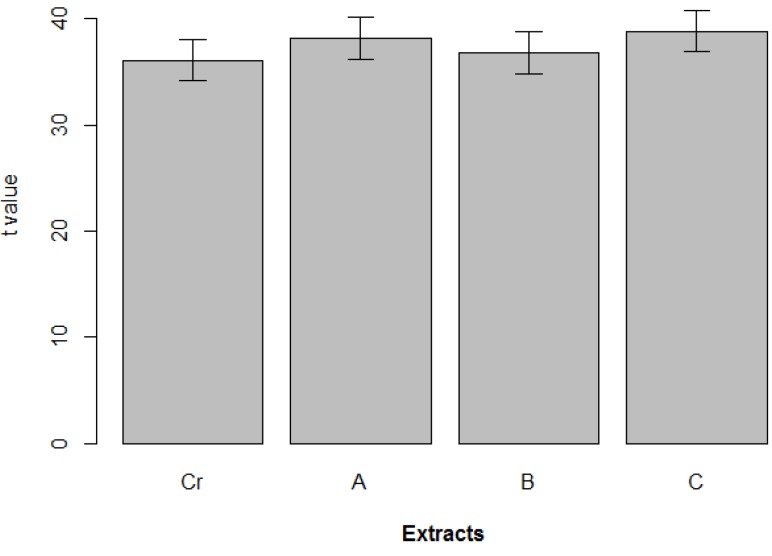
Global toxicity of the cyanobacterial crude extract (Cr) and fractions A, B and C. The average effect of extract on cell viability was calculated and standardized, as in Figure 3 for each extract (*n* ~ 6,003). Error bars show the standard error of the estimated difference.

The results obtained with the MTT assay are summarized in Table 1 and Figure 2, Figure 3 and Figure 4. This initial screening led to the identification of strains producing stronger cytotoxic effects on cancer cell lines. In Table 1, we present a classification of the cytotoxicity of each cyanobacteria strain globally, on each cell line. For each cell line, the strains were included in different classes: “strong cytotoxic”, “moderately cytotoxic”, “low cytotoxic” and “no cytotoxic”. These classes were established based on the percentiles of the distribution of the standardized average effect on cell viability, which were above the 90th percentile when standardized cell viability was less than 10%, between the 70th and 90th percentile when standardized cell viability was between 30% and 10% and between the 50th and 70th when standardized cell viability was between 50% and 30%, respectively. The distribution of this variable was assumed to be random normal. Standardization was performed with the *t* statistic.

**Table 1:** Summary of cell viability data from the MTT (3-(4,5-dimethylthiazol-2-yl)-2,5-diphenyl tetrazolium bromide) assays, after exposure to the cyanobacterial crude extract and A, B and C fractions. +++ indicates strong toxicity (higher than the 90th percentile of the effect on cell viability); ++ indicates moderate toxicity (the 70th–90th percentile of the effect on cell viability); + indicates low toxicity (the 50th–70th percentile of the effect on cell viability); − indicates no toxicity (less than the 50th percentile of the effect on cell viability). LEGE, Laboratory of Ecotoxicology, Genomics and Evolution.

Strain		Cancer Cell Lines							
		HepG2	RKO	MG-63	SK-BR-3	T47D	HT-29	SH-SY5Y	PC-3
*Nodosilinea nodulosa*	LEGE 06152	−	−	−	−	−	+++	+	+++
*Leptolyngbya* cf. *halophila*	LEGE 06102	+++	+	++	−	−	++	+	++
*Leptolyngbya mycoidea*	LEGE 06108	−	−	−	−	++	−	−	−
*Leptolyngbya mycoidea*	LEGE 06118	+	+	++	−	++	+	++	−
*Leptolyngbya mycoidea*	LEGE 06009	−	−	−	−	++	+++	+	++
*Leptolyngbya fragilis*	LEGE 07167	++	++	+++	++	+++	+	+++	+++
*Pseudanabaena* aff. *curta*	LEGE 07160	−	+	+	−	−	++	−	++
*Pseudanabaena* aff. *curta*	LEGE 07169	++	−	+++	+	++	+++	−	+++
*Pseudanabaena* aff. *persicina*	LEGE 07163	−	−	−	−	−	−	−	−
*Pseudanabaena* sp.	LEGE 06144	+++	−	−	+++	+	−	−	−
*Pseudanabaena* sp.	LEGE 06194	−	−	−	−	−	−	−	−
*Cyanobium* sp.	LEGE 06098	−	−	++	+	−	−	++	−
*Cyanobium* sp.	LEGE 06134	−	−	+	−	−	+	−	−
*Cyanobium* sp.	LEGE 07175	+	−	++	−	++	−	−	−
*Cyanobium* sp.	LEGE 07186	−	−	++	−	++	++	−	++
*Cyanobium* sp.	LEGE 06113	+++	−	+	++	−	−	++	−
*Cyanobium* sp.	LEGE 06137	−	++	−	−	−	−	+	−
*Cyanobium* sp.	LEGE 06097	+	−	−	−	++	+	−	−
*Cyanobium* sp.	LEGE 06139	−	++	−	−	+	−	+	−
*Synechococcus nidulans*	LEGE 07171	+	−	−	+++	−	−	++	−
*Synechococcus* sp.	LEGE 07172	−	++	−	−	−	−	++	−
*Synechococcus* sp.	LEGE 06005	−	−	++	−	−	−	−	−
*Synechococcus* sp.	LEGE 06026	−	−	−	+	−	+	−	−
*Synechocystis salina*	LEGE 06099	+++	−	++	+++	−	−	+	−
*Synechocystis salina*	LEGE 06155	+++	+++	+	++	+	+	++	−
*Synechocystis salina*	LEGE 07173	+	−	−	−	+	−	++	−
*Romeria* sp.	LEGE 06013	−	++	−	−	−	−	+	−
*Romeria* aff. *gracilis*	LEGE 07310	+	−	+	+++	++	−	++	−

**Figure 5:**
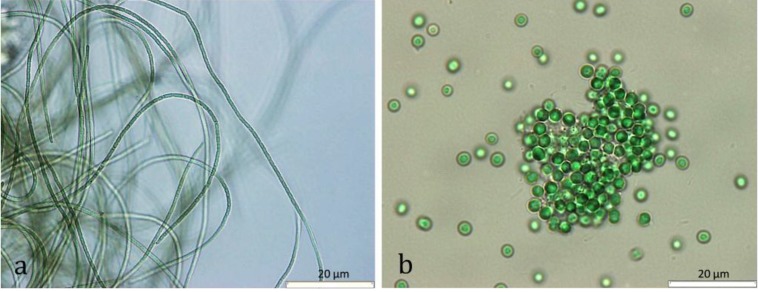
Micrographs of the cyanobacteria strains, *Leptolyngbya fragilis* LEGE 07167 (**a**) and *Synechocystis salina* LEGE 06155 (**b**).

Within this distribution, the effect of 8.9% of the strains was above the 90th percentile, which was considered a strong cytotoxic effect; 17.8% was between the 70th and 90th percentiles, which was considered as moderate cytotoxicity, and 14.3% appeared between the 50th and 70th percentiles, which was considered a low toxicity effect. The remaining 59% of strains were below the 50th percentile, considered as having no cytotoxic effect. However, the majority of the tested cyanobacterial strains were capable of inducing cytotoxicity in at least one of the cell lines (Table 1). In Figure 3 are shown the global results of the effect on cell viability for each cyanobacterial strain, extract and extract concentration, pooling all the cancer cell lines, times of exposure and individual replicates. In almost all strains, dark spots are present (the arrows indicate example) indicative of strong and moderate cytotoxic effects. Strain *Leptolyngbya fragilis* LEGE (Laboratory of Ecotoxicology, Genomics and Evolution) 07167 and *Synechocystis salina* LEGE 06155 (Figure 5) were the most cytotoxic strains (Figure 2 and Figure 3) with above the 90th percentile of the standardized effect on cancer cell viability, classified as a strong effect. Strains *Pseudanabaena* aff. LEGE 07163 and *Pseudanabaena* sp. LEGE 06194 were not found to induce cytotoxicity in any of the assays, representing only 7.2% of the cyanobacteria strains under study. Figure 4 shows that the crude extract was the most cytotoxic to the cancer cell lines, followed by fraction B. Fraction C was the one with the least bioactivity.

**Figure 6:**
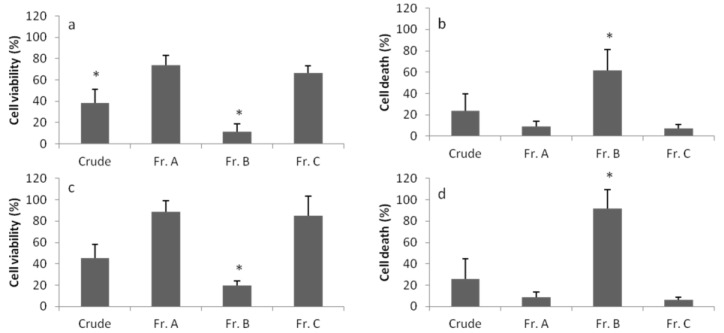
Cytotoxicity/cell viability induced by crude extract (Cr) and fractions (Fr.) of the *Synechocystis salina* LEGE 06155 on RKO and HepG2 cell lines, measured with MTT and LDH (lactic dehydrogenase release) assays. (**a**) RKO cell line, MTT assay; (**b**) RKO cell line, LDH assay; (**c**) HepG2 cell line, MTT assay; and (**d**) HepG2 cell line, LDH assay. * *p* < 0.001.

The assays where a strong cytotoxic effect was registered were additionally subjected to the LDH assay. As a comparison between MTT and LDH assays, Table 2 shows the fractions with higher cytotoxicity on each cell line for each of the strains tested. Extract B from the *Synechocystis salina* LEGE 06155 was the fraction with higher activity in both assays in HepG2 and RKO (Figure 6). Fraction A of the *Synechocystis salina* LEGE 06099 was also the most active fraction when tested in the HepG2 cell line. Furthermore, fraction A of the *Leptolyngbya cf. halophila* LEGE 06102 was the most active when tested with LDH and MTT assays.

**Table 2:** Summary of the results obtained with the two cytotoxic assays. The most active fractions are indicated and the concordant results highlighted in grey. The strains with no active fraction are marked with a hyphen.

Cell line	Strain	MTT	LDH
HepG2	LEGE 06155	B	B
	LEGE 06099	A	A
	LEGE 06102	A	A
	LEGE 06113	B	A
	LEGE 06144	Crude	-
HT-29	LEGE 07169	C	A
	LEGE 06009	Crude	-
	LEGE 06152	B	B
MG-63	LEGE 07169	B	-
	LEGE 07167	Crude, A, B, C	-
PC-3	LEGE 07169	A	-
	LEGE 06009	A	A
	LEGE 06152	Crude, A, B, C	-
RKO	LEGE 06155	B	B
SH-SY5Y	LEGE 07167	B	-
T47D	LEGE 07167	B	Crude

However, the activity shown by each fraction in the MTT assay was not always confirmed by the LDH assay. Fraction B of *Nodosilinea nodulosa* LEGE 06152 was the most active in both assays when tested in the HT-29 cell line. However, in PC-3, all the extracts showed activity when tested with the MTT assay, whereas this activity was not seen with the LDH. A similar profile was shown by *Leptolyngbya mycoidea* LEGE 06009, for which, when tested with both toxicological assays in PC-3, fraction A was the most active. In the HT-29 cell line, however, the crude extract showed the highest activity, but this activity was not detected with the LDH assay.

The strain, *Pseudanabaena* aff. *curta* LEGE 07169, appeared with a strong activity in several of the cell lines from this study: HT-29, MG63 and PC-3. However, the most active fraction detected by the MTT and LDH assays was not the same. Fractions A, B and C appear to induce cytotoxic effects with the MTT assay. When the LDH assay was applied, fraction A showed cytotoxicity in the HT-29 cell line, but for MG63 and PC-3 cell lines, the cytotoxic effect was not confirmed.

*Leptolyngbya fragilis* LEGE 07167, one of the two strains with a stronger activity, when tested with the MTT assay in SH-SY5Y and in T47D cell lines pointed out fraction B as the most active. In the MG63 cell line, all the fractions appeared to be strongly active. However, in the LDH assay, the crude extract appeared as the most active in the T47D cell line. In SH-SY5Y and MG63, no activity was detected.

## 3. Discussion

Picoplanktonic and filamentous marine cyanobacteria of genera *Nodosilinea*, *Cyanobium*, *Synechocystis*, *Synechococcus*, *Leptolyngbya*, *Pseudanabaena* and *Romeria* have been rarely studied with respect to their potential as producers of interesting bioactive compounds. In this study, we found that strains from those genera were able to induce cytotoxic effects in human cancer cell lines. Since these strains were not found to grow naturally in large densities, massive growth under laboratory conditions was necessary for biomass production.

Bioassays-guided fractionation is a methodology that has been successfully used in the isolation and identification of marine natural compounds, as it allows the quick recognition of the active fractions that may contain interesting compounds [21,22,23]. The search for new anticancer drugs has been currently associated with this approach [8,24,25]. In this work, cyanobacteria crude extract and fractions obtained by chromatography of twenty eight strains were tested in eight cancer cell lines, which were selected as being representative of several human tumors. By increasing the range of cell lines, we were able to increase the probability of obtaining cytotoxic effects.

Two of the most common cytotoxicity assays were applied in order to increase the consistency of results and to identify more precisely the potentially interesting cyanobacteria strains. When the results of both assays were compared, six cyanobacteria strains were shown to be the most interesting for the isolation of bioactive compounds: *Synechocystis salina* LEGE 06155 and LEGE 06099, *Leptolyngbya cf. halophila* LEGE 06102, *Leptolyngbya mycoidea* LEGE 06009, *Leptolyngbya fragilis* LEGE 07167 and *Nodosilinea nodulosa* LEGE 06152. The results from both assays did not match perfectly. This was already observed in other studies in which the MTT and LDH assays were tested together [26,27,28]. The MTT assay is clearly the most sensitive assay, since the rate of cytotoxicity caused by the cyanobacteria extracts observed with this assay was generally higher than that observed with the LDH assay. This can be explained by the fact that each assay is based on different cellular events. The lactate dehydrogenase assay is based on the release of the enzyme into the culture medium after cell membrane damage. Thereby, the assay is satisfactory when agents that induce cell membrane damage are involved in the mechanism. Certain cytotoxic metabolites could cause cell alterations only in intracellular activities or organelles and do not affect the membrane; in those cases, the toxicity would not be detected [29]. The MTT assay is based on the enzymatic reduction of the 3-(4,5-dimethylthiazol-2-yl)-2,5-diphenyl tetrazolium bromide (MTT) into a blue colored formazan in the mitochondria. As with the LDH, some cytotoxic metabolites could be missed, since the metabolite could affect some cell organelles without interfering with the mitochondrial functions [29]. In addition, cytotoxic events can occur with different timing, initially affecting the mitochondria and, later, the membrane.

Still, considering both MTT and LDH results and using a bioassay-guided fractionation, HepG2 and RKO cell lines could be used to isolate a possible compound(s) present in fraction B of *Synechocystis salina* LEGE 06155, as this was one of the two strains inducing stronger toxicity. Fractions A of *Leptolyngbya cf. halophila* LEGE 06102 and *Synechocystis salina* LEGE 06099 also have interest for the isolation of an anticancer compound using the HepG2 cell line. Fraction B of *Nodosilinea nodulosa* LEGE 06152 and fraction A of *Leptolyngbya mycoidea* LEGE 06009 can also be explored using HT-29 and PC-3, respectively.

The strains, *Leptolyngbya fragilis* LEGE 07167 and *Synechocystis salina* LEGE 06155, were shown to be the most bioactive in the tested cancer cell lines. Several bioactive compounds with anticancer properties were already isolated from *Leptolyngbya* genera. Coibamide A, isolated from a *Leptolyngbya* strain collected in Coiba National Park (Coiba Island, Panama), demonstrated anticancer activity against lung cancer NCI-H460, breast cancer MDA-MB-231, melanoma LOX IMVI, leukemia HL-60 and astrocytoma SNB75 [12]. Dolastatin 12, isolated from a strain collected in the Red Sea, was cytotoxic to mouse neuro-2a blastoma cells [24]. From *Synechocystis* genera, several fatty acids, volatile compounds and pigments were already isolated [30], but to the best of our knowledge, any bioactive anticancer compound was already identified.

Considering the MTT screening assay, all but two of the selected cyanobacteria strains revealed bioactivity. However, in many cases, the cytotoxic effect was evident only for the 100 μg·mL^−1^ concentration. This concentration was already demonstrated to be the most effective in a screening performed by Leão and co-workers using likewise marine cyanobacteria extracts and testing them in several ecologically-relevant bioassays [31]. Previous similar cytotoxicity screenings with terrestrial and freshwater cyanobacteria strains also showed positive results in a large portion of extracts, although not so strong as in this study [32,33,34].

Crude extract was globally the most bioactive. This finding can be explained by the presence of a cocktail of bioactive compounds, since all the compounds produced by the cyanobacteria and then fractionated into fractions A, B and C would be included in this extract. Fraction B is the second most active. It contains the compounds with intermediate polarity, since it was eluted from the column with the solvents with intermediate polarity (a higher percentage of ethyl acetate). This fraction usually contains several classes of peptides and depsipeptides. In fact, many compounds belonging to these chemical classes were already described and isolated from marine cyanobacteria and demonstrated to have anticancer potential [7,10,35]. In some cases, like the strain, *Pseudanabaena* aff. *curta* LEGE 07169, more than just one fraction was shown to be bioactive. This could be explained by the presence of the same compound in adjacent fractions, since the fractions are removed gradually from the column, and/or by the presence of more than one compound with anticancer properties.

Some strains of the genera included in this study, namely *Cyanobium*, *Synechocystis*, *Synechococcus* and *Leptolyngbya*, were already identified as a potential source of bioactive compounds, based on screenings with mammals [36], invertebrates [37,38] virus, bacteria and some cell lines [39,40], although only compounds from *Leptolyngbya* were yet isolated [12,24]. The results obtained in the present study allow us to confirm the interest of this picoplanktonic and filamentous genera as a source of anticancer compounds.

## 4. Experimental Section

### 4.1. Cyanobacteria Strains and Culture

**Figure 7:**
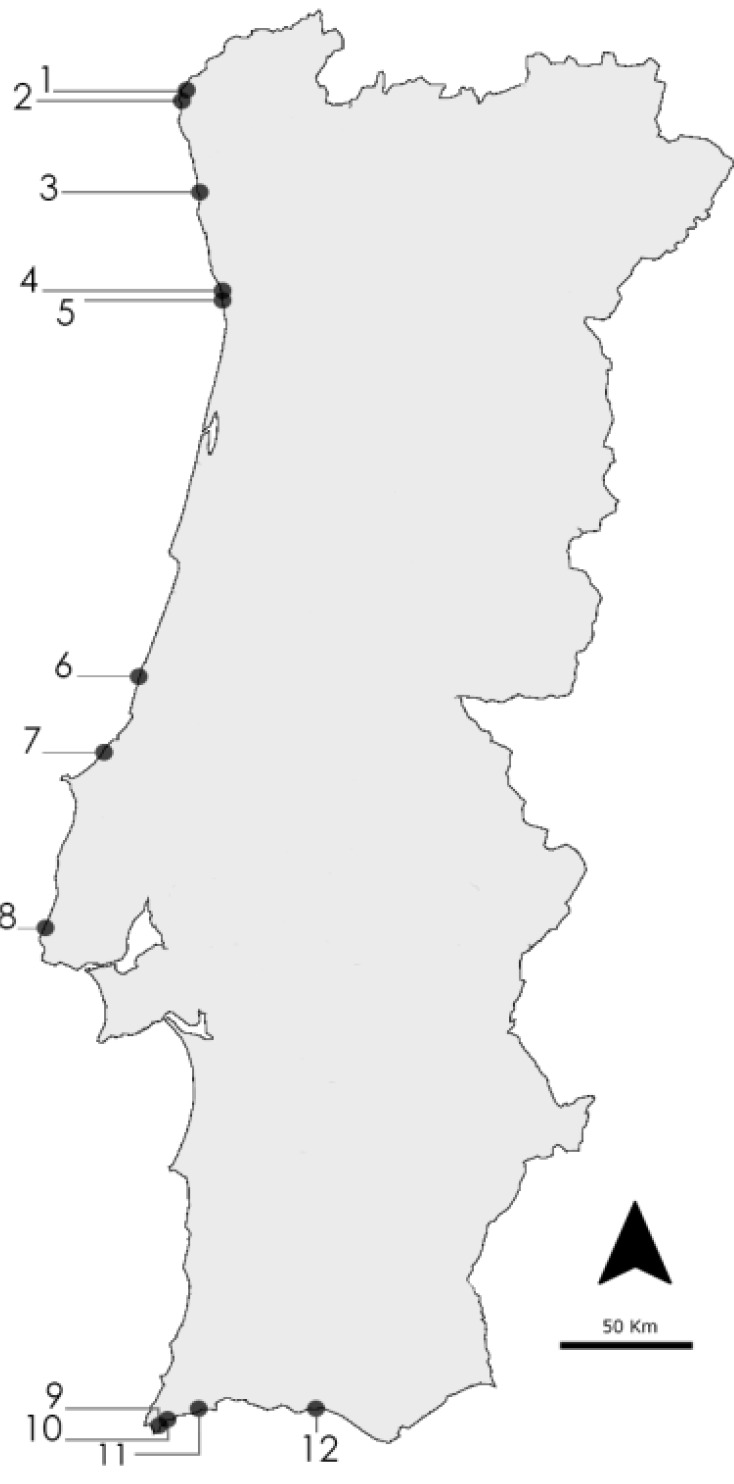
Location of the rocky beaches from where cyanobacteria strains were isolated. The numbers correspond to rocky beaches, referring to the sampling location in Table 3.

Twenty eight marine cyanobacterial strains belonging to the coccoid genera, *Cyanobium*, *Synechocystis* and *Synechococcus*, and the filamentous genera, *Nodosilinea*, *Leptolyngbya*, *Pseudanabaena* and *Romeria*, were employed in this study (Table 3). Strains were isolated from the Portuguese coast (Figure 7) and are maintained in the LEGE (Laboratory of Ecotoxicology, Genomics and Evolution, (Porto, Portugal) culture collection. Large-scale cultures of these strains were set for biomass production. Strains were grown in Z8 medium [41], supplemented with 20 g·L^−1^ NaCl, or in MN medium [42] (Table 3). Cultures were maintained at 25 °C, with a light intensity of 10 μmol photons m^−2^·s^−1^ and with a light/dark cycle of 14:10 h. At the exponential growth phase, cells were harvested by centrifugation, frozen at −20 °C and freeze-dried. The lyophilized biomass was stored at −20 °C.

**Table 3:** Marine cyanobacteria strains included in this study, origin, accession number and culture medium.

Taxon	Code	Sampling Location	Accession Number	Reference	Medium
*Nodosilinea nodulosa*	LEGE 06152	Lavadores (4)	HQ832915	[31]	Z8
*Leptolyngbya cf. halophila*	LEGE 06102	S. Bartolomeu do Mar (2)	HQ832906	[43]	Z8
*Leptolyngbya mycoidea*	LEGE 06108	Luz (11)	HQ832942	[43]	Z8
*Leptolyngbya mycoidea*	LEGE 06118	Luz (11)	HQ832943	[43]	Z8
*Leptolyngbya mycoidea*	LEGE 06009	Foz do Arelho (7)	JF708121	[43]	Z8
*Leptolyngbya fragilis*	LEGE 07167	Lavadores (4)	HQ832917	[43]	MN
*Pseudanabaena* aff. *curta*	LEGE 07160	Olhos d’Água (12)	HQ832948	[43]	MN
*Pseudanabaena* aff. *curta*	LEGE 07169	Aguda (5)	HQ832923	[43]	MN
*Pseudanabaena* aff. *persicina*	LEGE 07163	Moledo (1)	HQ832900	[43]	MN
*Pseudanabaena* sp.	LEGE 06144	Burgau (10)	HQ832937	[43]	MN
*Pseudanabaena* sp.	LEGE 06194	Luz (11)	-	-	MN
*Cyanobium* sp.	LEGE 06098	Martinhal (9)	KC469572	[44]	Z8
*Cyanobium* sp.	LEGE 06134	Moledo (1)	KC469573	[44]	Z8
*Cyanobium* sp.	LEGE 07175	Martinhal (9)	KC469575	[44]	Z8
*Cyanobium* sp.	LEGE 07186	Martinhal (9)	KC469576	[44]	Z8
*Cyanobium* sp.	LEGE 06113	Aguda (5)	KC469577	[45]	Z8
*Cyanobium* sp.	LEGE 06137	Lavadores (4)	HQ832914	[43]	Z8
*Cyanobium* sp.	LEGE 06097	Martinhal (9)	HQ832928	[43]	Z8
*Cyanobium* sp.	LEGE 06139	Aguda (5)	KC469574	[44]	Z8
*Synechococcus nidulans*	LEGE 07171	Burgau (10)	HQ832939	[43]	Z8
*Synechococcus* sp.	LEGE 07172	Olhos d’Água (12)	HQ832950	[43]	Z8
*Synechococcus* sp.	LEGE 06005	São Pedro de Moel (6)	HM124558	[37]	Z8
*Synechococcus* sp.	LEGE 06026	Empa (8)	-	-	Z8
*Synechocystis salina*	LEGE 06099	Moledo (1)	HQ832895	[43]	Z8
*Synechocystis salina*	LEGE 06155	S. Bartolomeu do Mar (2)	HQ832911	[43]	Z8
*Synechocystis salina*	LEGE 07173	Olhos d’Água (12)	HQ832951	[43]	Z8
*Romeria* sp.	LEGE 06013	Foz do Arelho (7)	HQ832927	[43]	Z8
*Romeria* aff. * gracilis*	LEGE 07310	Minho estuary (3)	HM217057	[46]	Z8

### 4.2. Extract Preparation

A crude organic extract was obtained by repeated extraction of approximately 1 g (dry weigh) of cyanobacterial biomass. Extraction was performed with CH_2_Cl_2_:MeOH (2:1) at room temperature and 40 °C. This crude extract was fractioned by vacuum liquid chromatography, by injection in SiOH normal phase SPE cartridge (Strata SI-1, Phenomenex), using a gradient elution from hexane-EtOAc-MeOH, yielding three fractions, hereafter A, B and C, respectively. The crude extract and fractions were solubilized in dimethyl sulfoxide (DMSO).

### 4.3. Cell Lines

Human colon adenocarcinoma (HT-29), neuroblastoma (SH-SY5Y) and breast carcinoma cell lines T47D were purchased from Sigma-Aldrich. PC-3 prostate adenocarcinoma, SK-BR-3 breast adenocarcinoma, RKO colon carcinoma, hepatocellular carcinoma HepG2 and MG-63 osteosarcoma cell lines were obtained from the American Type Culture Collection (ATCC). Cell lines were cultivated in modified Eagle medium (DMEM Glutamax), except PC-3, which was grown in minimal essential medium (α-MEM), and SH-SY5Y, grown in HAM F12 Medium. DMEM Glutamax and α-MEM mediums were supplemented with 10% fetal bovine serum (FBS), 2.5 μg·mL^−1^ fungizone, penicillin-streptomycin (100 IU·mL^−1^ and 100 μg·mL^−1^, respectively). HAM F12 medium was supplemented with 2 mM glutamine, 1% non-essential amino acids and 15% FBS. Cells were incubated in a humidified atmosphere with 5% of CO_2_, at 37 °C. Culture medium was renewed every two days. At 80%–90% cell confluence, adherent cells were enzymatically released with a solution of 0.05% trypsin in 0.25% EDTA.

### 4.4. Cytotoxicity Assays

#### 4.4.1. MTT Assay

Cellular viability was evaluated by the reduction of the 3-(4,5-dimethylthiazole-2-yl)-2,5-diphenyl tetrazolium bromide (MTT, Sigma-Aldrich, St. Louis, MO, USA). All cell lines were seeded in 96-well culture plates at 10^4^ cells·cm^−2^, except SH-SY5Y, which were seeded at 3.1 × 10^4^ cells·cm^−2^. Cells adhesion was allowed during 24 h. Then, cells were incubated in new medium with the crude extract and fractions at 100, 10 and 1 μg mL^−1^ and 1% DMSO as the negative control during 24, 48 and 72 h. After treatments, cells were incubated 4 h, at 37 °C, with 0.05 mg mL^−1^ MTT. The purple colored formazan salts formed were dissolved in DMSO, and the absorbance was read at 550 nm in a GEN5TM-Multi-detection Microplate Reader (Biotek, Bad Friedrichshall, Germany). All assays were run in triplicate and averaged. Cytotoxicity was expressed as a percentage of cell viability considering 100% viability in the negative control (cells treated with 1% DMSO).

#### 4.4.2. LDH Release Assay

The LDH release was assessed using the *In Vitro* Toxicology Assay Kit, Lactic Dehydrogenase-based (TOX7, Sigma-Aldrich), according to the manufacturer’s instructions. Cell lines were seeded in 96-well culture plates at 3.1 × 10^4^ cells cm^−2^. After 24 h of adhesion, cells were exposed to new medium with the crude extract and fractions at 100 μg mL^−1^ for 24, 48 and 72 h. A positive control, leading to 100% cytotoxicity by lysing the cells completely, was included in the assay.

The LDH values were measured at 490 nm, with 690 nm as the reference in a GEN5™-Multi-detection Microplate Reader (Biotek). All the assays were run in triplicate and averaged. The amount of LDH leakage to the medium was calculated according to the formula:

LDH leakage (%) = LDH medium/total LDH × 100
(1)

### 4.5. Statistical Analysis

Our main approach to analyze data was using general linear models. A single analysis was performed for each cyanobacterial strain. The percentage of viability was used as the dependent variable. The extract, concentration and time of incubation were used as fixed factors. If any of these factors was not significant, it was removed from the model.

Normality was tested in the model residuals with the Shapiro–Wilk test. In those cases where residual distribution was not normal, the data were normalized. In order to do so, preferably, the data were transformed using the Box-Cox function. If this first approach did not work, outliers with a Cook distance >0.5 were removed. After outlier removal, data might be transformed again with the Box–Cox function, if needed. The number of outliers removed, if any, never exceeded 1% of the total data. If this approach combining outlier removal and data transformation failed, a generalized linear model was employed, with the gamma distribution as the error distribution function. In these cases, the goodness of fit to gamma distribution was tested in the dependent variable with a *χ*^2^ test. The vast majority of the analyses were, however, general linear models. *Post hoc* pairwise comparisons among factor levels were performed with the Tukey test. Homogeneous subsets of treatment levels were obtained based on the significance levels of the Tukey pairwise comparisons. Those levels that did not differ significantly among them were classified into the same subset.

The software employed was R version 2.15.2 (R Foundation for Statistical Computing, Vienna, Austria) with functions from the base, stats, car, multcomp and nlme packages.

## 5. Conclusions

Picoplanktonic marine cyanobacteria of genera *Cyanobium*, *Synechocystis*, *Synechococcus* and filamentous forms of the genera, *Nodosilinea*, *Leptolyngbya*, *Pseudanabaena* and *Romeria*, isolated from the Portuguese coast, revealed high potential as a source of anticancer compounds. By performing an intensive screening applying the two most common cytotoxicity assays, MTT and LDH, we were able to select six cyanobacteria strains, from an initial pool of twenty-eight, as interesting for the isolation of compounds with potential anticancer activity. The identification of new sources of natural products is an important step in drug discovery. In this sense, the results from this screening highlight the potential of these marine cyanobacteria genera as a source of interesting bioactive compounds.
